# Identification and optogenetic manipulation of memory engrams in the hippocampus

**DOI:** 10.3389/fnbeh.2013.00226

**Published:** 2014-01-17

**Authors:** Steve Ramirez, Susumu Tonegawa, Xu Liu

**Affiliations:** ^1^Department of Biology and Department of Brain and Cognitive Sciences, RIKEN-MIT Center for Neural Circuit Genetics at the Picower Institute for Learning and Memory, Massachusetts Institute of TechnologyCambridge, MA, USA; ^2^Howard Hughes Medical Institute, Massachusetts Institute of TechnologyCambridge, MA, USA

**Keywords:** optogenetics, memory engram, IEG, ChR2, false memory

## Abstract

With the accumulation of our knowledge about how memories are formed, consolidated, retrieved, and updated, neuroscience is now reaching a point where discrete memories can be identified and manipulated at rapid timescales. Here, we start with historical studies that lead to the modern memory engram theory. Then, we will review recent advances in memory engram research that combine transgenic and optogenetic approaches to reveal the underlying neuronal substrates sufficient for activating mnemonic processes. We will focus on three concepts: (1) isolating memory engrams at the level of single cells to tag them for subsequent manipulation; (2) testing the sufficiency of these engrams for memory recall by artificially activating them; and (3) presenting new stimuli during the artificial activation of these engrams to induce an association between the two to form a false memory. We propose that hippocampal cells that show activity-dependent changes during learning construct a cellular basis for contextual memory engrams.

## A biological locus for memory

Memories thread and unify our overall personal narrative. Disruption of the putative neural correlates for memories in humans leads to devastating maladies and dramatically impairs cognition. Even when they are not subject to experimenter manipulations or natural insults, memories are not fully veridical representations of past experiences. Recalling a memory makes it labile, which can distort the mental representation of an event, incorporate misinformation, and sometimes even fabricate illusory episodes entirely (Schacter and Loftus, 2013). Despite the importance of memories in our daily lives and the comprehensive studies on this subject, the process by which memories emerge through the interactions of neurons distributed across various brain regions is a poorly understood phenomenon (Eichenbaum, 2004; Squire et al., 2007).

The biological conceptualization of a memory was given a name—an “engram”, by the German Zoologist Richard Semon in 1921 (Semon, 1921). A decade later, the American Psychologist Lashley et al. (1932) pioneered a systematic hunt for engrams in the rodent brain by lesioning various parts of cerebral cortex and relating the size and the location of the lesion to behavioral performance on a maze task. His experiments lead him to formulate the *mass action principle*, which posits that memories are spread throughout the cortex and not localized to discrete brain regions (Lashley et al., 1932). For Lashley, the biological locus for a single memory remained elusive. Years later, the Canadian Neurosurgeon Penfield and Rasmussen (1950) observed the first tantalizing hint that certain memories could be localized in defined brain regions. During his surgeries for patients with epilepsy, Penfield applied small jolts of electricity to the brain to reveal which regions were centers for causing seizures. Remarkably, while stimulating parts of the medial temporal lobe (MTL), he observed that 8% of his patients reported vivid recall of random episodic memories (Penfield and Rasmussen, 1950). This finding suggests that the MTL region harbors the biological locus for episodic memory.

Then in 1953, the American Neurosurgeon William Scoville and British Neuropsychologist Brenda Milner tested the conjecture that the MTL had distinct contributions to episodic memories (Scoville and Milner, 1957). To treat the epileptic convulsions that incapacitated his patient Henry Molaison (H.M.), Scoville and Milner (1957), like Penfield and Rasmussen (1950), resected the problematic neural tissue, which involved removal of large sections of the hippocampus and adjacent areas in this case. For the decades to come, H.M. lost his ability to form new memories—to bridge personal events across large spans of time (anterograde amnesia)—while simultaneously failing to recall events for years leading up to his surgery (retrograde amnesia). Scoville and Milner (1957) work on H.M. also pointed to the MTL in general and to the hippocampus in particular as an essential locus for episodic memory.

Since then, a large number of subsequent studies in humans (Rempel-Clower et al., 1996; Schmolck et al., 2002), as well as in non-human primates and rodents (Jarrard, 1993; Zola and Squire, 2001), have established that the hippocampus is crucial for the formation of memories that include “what-where-when” components, or context-temporal-informational domains, which are known as episodic memories (Eichenbaum, 2004). Additionally, the hippocampus’ structure and function has been extraordinarily conserved across mammalian clades, permitting a thorough experimental interrogation and deconstruction of its functions in animal models (Eichenbaum, 2003).

As a candidate mechanism supporting mnemonic processes, the strengths of synapses throughout the hippocampus are thought to be altered in an experience-dependent manner. The idea of neural plasticity dates back to Plato, who originally conjectured that memories leave a stamp or trace in the mind analogous to the impression on a wax tablet left by a signet ring (Campbell, 1883). In the 21^st^ Century, Hebb (1949) put a modern spin on Plato’s dialogue and hypothesized that neurons that “fire together” also “wire together” (Hebb, 1949)—a conceptual antecedent of long-term potentiation (LTP). Bliss and Lomo (1973) experimental demonstration of LTP (Bliss and Lomo, 1973), followed by the essential role of NMDA receptors in LTP induction (Collingridge et al., 1983) opened a way to investigate LTP as synaptic mechanism underlying certain forms of learning and memory. The result of the initial pharmacological blockade experiments conducted with an NMDA receptor (NMDAR) antagonist, AP5, were consistent with the notion that LTP is essential for spatial learning (Morris et al., 1986), and the validity of this notion was demonstrated with the more definite targeted genetic ablation of the NMDAR in the CA1 region of the hippocampus (Tsien et al., 1996), although the cortical NMDAR may also have contributed to the phenotype (Fukaya et al., 2003). A subsequent report concluded that CA1 NMDAR were dispensable for spatial learning *per se*, but the same report showed an easily detectable level of NMDAR RNA in CA1 and hence the possibility that the remaining CA1 NMDAR supported spatial learning cannot be excluded (Bannerman et al., 2012). The authors reactivated an old hypothesis (Vinogradova, 1975) that ascribes CA1/DG NMDAR a different role in learning and memory processes. However, this provocative and controversial hypothesis would require careful and critical examination and further investigation in the future. Mice with NMDAR ablation in DG and CA3 showed impairments of pattern separation and pattern completion, respectively (McHugh et al., 1996, 2007; Nakazawa et al., 2003). Another study did not detect the effect of NMDAR deletion in the DG cells on pattern separation (Niewoehner et al., 2007), but this may be due to the fact that different behavioral paradigms were used. Overall, these observations supported the role of NMDAR-dependent synaptic plasticity, including LTP, in hippocampal-dependent memory.

## Molecular signatures of memories

In more recent years, the role of the different circuits within the hippocampal-entorhinal cortex network, as well as young vs. old DG granule cells, in specific aspects of hippocampal-dependent learning and memory has been identified by using targeted genetic manipulations (Nakashiba et al., 2008, 2009, 2012; Clelland et al., 2009; Drew et al., 2010; Suh et al., 2011). While these past studies have been pivotal in our understanding of the role of the different subfields and circuits in learning and memory, they were conducted without distinguishing between cells that were activated by specific sensory or cognitive stimuli and cells that remained inactive.

To identify which cells are active during the formation of a memory, one can rely on the activity-dependent nature of immediate early genes (IEGs). It is believed that the formation of long-term memory (LTM) requires gene transcription and protein translation at the time of training to alter neural morphology, receptor densities, and overall excitability of the cells (Jones et al., 2001). Multiple rounds of transcription have been identified after learning and most studies focus on IEGs, which are transcribed within minutes in an experience-dependent manner by transcription factor proteins already present in the cytoplasm of a neuron (Guzowski, 2002).

The most well characterized IEGs are* zif268*, *c-fos*, and *Arc/Arg3.1*, and all of them have been implicated in supporting memory formation. Mice with a deletion of *zif268* show deficits in contextual fear conditioning (CFC) and the hidden platform variant of the Morris Water Maze (MWM) test (Jones et al., 2001). A similar result was obtained in mice lacking the *c-fos* gene in the central nervous system (Fleischmann et al., 2003). These mice also had impaired LTP, but the developmental effects of *c-fos* gene deletion could not be excluded from contributing to the observed phenotypes. Post-developmental antisense oligodeoxynucleotide (As-ODN)-mediated blockade of *c-fos* translation in the hippocampus caused impaired consolidation of inhibitory avoidance, MWM, and socially transmitted food preference behaviors (Guzowski and McGaugh, 1997; Guzowski, 2002; Countryman et al., 2005), all of which are tasks thought to depend on the integrity of the hippocampus. Translational inhibition of *Arc* by As-ODN, and mice with global genetic deletion of *Arc*, have demonstrated an obligatory role for this IEG in memory consolidation for MWM, fear conditioning, conditioned taste aversion, and novel object recognition tasks (Guzowski et al., 2000; Plath et al., 2006).

In addition, studies of *Arc* and *c-fos* expression after behavioral training have shown that the proportion of cells expressing these IEGs in DG (2–6%), CA3 (20–40%), and CA1 (40–70%) after exposure to a novel environment resembles the proportion of hippocampal excitatory cells physiologically active in a given environment, which further validates the use of IEGs as an indicator of recent neural activity (Vazdarjanova and Guzowski, 2004). The cellular expression pattern of *c-fos* and *Arc* is different for different contexts, but remains stable upon re-exposure to the same context. These observations indicate that memory engram is highly conjunctive in nature but with remarkably labile synaptic properties that allow flexible memory updating (Guzowski et al., 2006; Richards and Frankland, 2013). It is in light of these studies that our first hypothesis for memory engrams emerges—namely, cells expressing *c-fos* after a training episode are participating in the encoding of the memory for that specific experience. Therefore, these cells may represent a component of the stored memory engram.

## Experimental evidence for memory engrams

To pinpoint a biological process as the underlying mechanism for a specific phenomenon, three types of evidence are normally required. These are: correlation, blockade, and mimicry. Correlation is to record the parallel occurrence between the phenomenon and the process, which will show an indirect relationship between these two; blockade means interrupting the candidate process, and if this also interferes with the phenomenon, then this shows the *necessity* of the process for the expression of the phenomenon; mimicry is to artificially generate the process, and if by doing so one can recreate the phenomenon, then this demonstrates *sufficiency*.

These principles also apply if one wants to demonstrate that engram-bearing cells are the basis for memories. For correlation experiments, molecular and physiological changes were found in specific neuronal ensembles accompanying memory formation from insects to humans. In the *Drosophila* olfactory learning circuit, defined neuronal populations or even single neurons change their response properties selectively towards odors used in training after olfactory conditioning (Yu et al., 2006; Liu and Davis, 2009). In mice, overlapping populations of cells in the amygdala are activated during the acquisition and recall of a fear memory (Reijmers et al., 2007). IEGs are expressed in largely overlapping populations of neurons in the rat hippocampus and neocortex during repeated exposure to the same environment (Guzowski et al., 1999; Vazdarjanova et al., 2002). In addition, single neurons recorded from the human hippocampus and MTL are shown to respond reliably to the same images or episodes (Quiroga et al., 2005; Gelbard-Sagiv et al., 2008). These observations suggest that, if there is a cellular basis for memory across different species, then it is sparsely encoded in a stable population of neurons.

Researchers have also conducted blockade experiments on selected cells to show the necessity of engram-bearing cells for different types of memories. In two studies, researchers pioneered a novel loss-of-function approach to perturb a component of a memory engram. By selectively ablating or inhibiting sparse population of cells in the amygdala that are preferentially recruited into the representation of a fear memory, the researchers interfered with the recall of that memory in mice (Han et al., 2009; Zhou et al., 2009). Moreover, in rats, selective inactivation of a small population of neurons in the nucleus accumbens that were previously activated by cocaine has also been shown to attenuate the memory for the drug-associated environment (Koya et al., 2009).

Compared to the observation and blockade experiments, the mimicry experiments for memory engram studies remained a considerable challenge. Although it had been widely recognized and agreed that such experiments are essential to test the engram hypothesis (Martin and Morris, 2002; Gerber et al., 2004), the lack of tools that could precisely label and control selected neurons involved in a particular memory posed a formidable obstacle to carry out these experiments. Demonstrating the existence of memory engrams at the cellular level requires a system that can selectively label and activate the memory engram-bearing cells to induce the predicted behavioral changes caused by learning.

## Identifying memory engrams

To selectively activate a cell population bearing an engram for a particular memory, one needs to be able to isolate and label these cells for future manipulation. Our first goal was to develop and characterize an activity-dependent and inducible system to label only the cells involved in the formation of a specific memory with channelrhodopsin-2 (ChR2). ChR2 is a light-sensitive channel that allows the influx of cations when illuminated by ~470 nm blue light (Nagel et al., 2003), resulting in the activation of the neurons expressing this channel. We began by expressing ChR2-EYFP fusion protein in an activity dependent, doxycycline (Dox)-regulated manner. This approach ensures that only neurons active during a defined episode become labeled for subsequent control by light stimulation.

**Figure 1:**
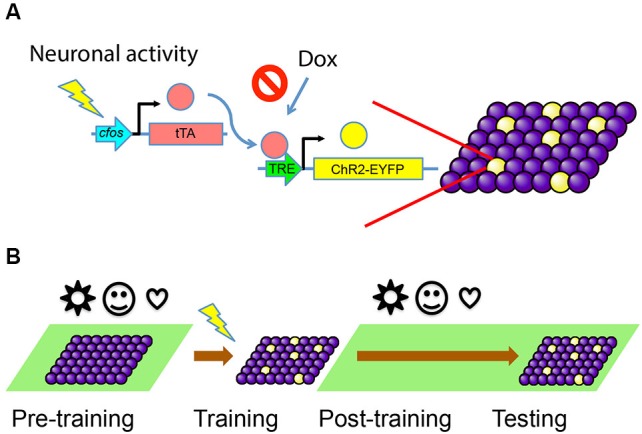
**Activity-dependent labeling of neurons. (A)** Training-induced neuronal activity drives the expression of tTA, which in turn activates downstream gene expression through TRE promoter and labels active cells with ChR2-EYFP (yellow). This process can be blocked by the presence of Dox. **(B)** The animals are kept on Dox (green background) before the training, so any irrelevant stimuli (represented by various symbols) during this period will not cause cells to express ChR2-EYFP. The animals are taken off Dox during training, so cells active during the memory formation will be labeled. Animals are put back on Dox diet post-training and during testing, so no further labeling happens.

We used the TetTag mouse (Reijmers et al., 2007), which harbors a pivotal transgene of interest, *c-fos*- tetracycline transactivator (tTA). This transgene contains the *c-fos* promoter, which drives the expression of the tTA. In this transgenic line, tTA can mimic the expression pattern of endogenous *c-fos* and only transiently appear in activated cells. The tTA protein will bind to the tetracycline-responsive element (TRE) to trigger the expression of a downstream target gene. The binding of tTA to TRE is blocked by Dox, which can be administered through an animal’s diet. If Dox is removed from the food, a window for activity-dependent labeling is opened and tTA can bind to TRE to turn on the expression of a gene of interest—ChR2-EYFP in this case—only in activated cells (Figure 1A).

Consistent with its role in processing various aspects of spatial and temporal information, the hippocampus is known to be critical for the formation of the contextual component of fear memories (Kim and Fanselow, 1992; Phillips and Ledoux, 1992). Of the various hippocampal subregions, computational models predict that DG in particular orthogonalizes inputs from entorhinal cortex into separate spatial representations (Treves and Rolls, 1994). Behavioral data support DG’s essential role in discriminating between similar contexts (McHugh et al., 2007), and *in vivo* recordings in freely moving animals show that DG granule cells are exquisitely sensitive to subtle changes in contextual information (Leutgeb et al., 2007). Cellular studies of IEG expression show that sparse populations of DG granule cells (2–6%) are activated in a given context (Chawla et al., 2005; Schmidt et al., 2012). Moreover, whereas a largely overlapping population of DG granule cells is activated repeatedly in the same environment, different environments or even different tasks in the same environment activate different populations of DG (Kubik et al., 2007; Satvat et al., 2011). These lines of evidence point to the DG as an ideal target for the formation of contextual memory engrams that represent discrete environments, guiding us to select the DG as our target for potential engram cell labeling.

We targeted the DG of *c-fos*-tTA transgenic mice with an AAV virus vector carrying TRE-ChR2-EYFP and implanted an optical fiber directly above the site of injection for light delivery. The mice were raised on a Dox diet to prevent any tTA-dependent transcription. This ensures that tTA produced by unintended activity throughout development or other experiences prior to our behavioral training will not induce the expression of ChR2-EYFP. When the mice are fear conditioned while on a Dox-free diet, the tTA expresses in a similar pattern as *c-fos* and enables the transcription of ChR2-EYFP, thus tagging putative fear engram-bearing cells. Then the mice are put back on diet containing Dox right after training to prevent the labeling of new cells in response to experiences after training (Figure 1B).

## Activating memory engrams

Prior to fear conditioning, we performed habituation sessions to measure the animals’ basal freezing levels to a novel context (context A), during which they were on Dox diet and ChR2-EYFP was not expressed. As expected, the animals showed minimal amounts of freezing behavior during both light-on and light-off epochs. They were then taken off Dox to open a window for activity-dependent labeling and fear conditioned in a different context (context B) to label fear memory engram-bearing cells, and placed back on Dox diet immediately after training. A day later, the animals were placed back into context A and stimulated with light to activate the neurons labeled during fear conditioning in context B and test the behavioral consequences.

This experiment directly tests the hypothesis that the *c-fos*-expressing cells in the hippocampus activated during training are sufficient for memory recall. Indeed, optogenetic reactivation of these cells resulted in freezing behavior indicative of fear memory recall (Liu et al., 2012), thus demonstrating their causal contributions to activating the behavioral expression of a memory.

Two control groups were also tested, and none of them showed any light induced freezing before or after training. The first control group did not receive foot shocks during the training. Histological data suggested that simply exposing mice to a novel context elicited as much DG activity as exposure to a novel context plus shock, as similar numbers (~6%) of DG cells became *c-fos* positive after either condition. This group demonstrated that the freezing in the experimental groups was not due to the optical activation of a population of hippocampal neurons unrelated to a fear memory. The second control group underwent the exactly same training as the experimental group but expressed EYFP alone instead of ChR2-EYFP. This control demonstrated that the light-induced freezing in the experimental groups was not due to changes in the salience of light after fear conditioning or other non-specific effects induced by light.

We also tested whether the light-activated memory recall was context-specific. We began by exposing animals that were off Dox to context A and label DG cells with ChR2-EYFP, then the animals were placed back on Dox to prevent any further labeling. They were then fear conditioned the next day in context B. Thus, these animals have both a ChR2-labeled non-fear memory engram for neutral context A and an unlabeled fear memory engram for context B. Crucially, our neuronal data showed that two statistically independent populations of DG cells were recruited to encode two discrete environments. These observations argue that DG orthogonalizes input at the neuronal ensemble level and recruits distinct sets of cells for distinct experiences. As predicted, this group of animals did not show increased freezing upon light stimulation, despite the presence of a fear memory from context B. This result argues that DG memory engram cell populations are context-specific.

Together, these data suggest that re-activating DG cells that were active during fear conditioning training is sufficient to induce the recall and behavioral expression of that fear memory. Accordingly, we propose that these cells form a cellular basis of a memory engram, and that two different contexts are parsed out as independent experiences represented by independent neuronal ensembles in DG.

Our study provides a methodological framework to study how an animal’s environment is represented in neuronal ensembles in the hippocampus. The strength of our system lies in its precision of tagging only relevant cells for future manipulation. This system can target specific brain regions, specific cell types, and also specific cell populations involved in a particular memory, which are otherwise indistinguishable from their neighboring cells. It focuses the tremendous power of optogenetics onto behaviorally relevant cell ensembles and enables the circuit and functional mapping of multiple memory engrams throughout the brain.

## False memories in the brain

In the early 1930s, the British psychologist Frederic Bartlett constructed and recited short but slightly inconsistent fables, most famously *The War of the Ghosts,* to test subjects from various cultural backgrounds (Bartlett, 1932). Strikingly, when asked to recall the fable, many subjects unknowingly modified the fable into a logical story that also contained new elements that fit within their cultural milieu. Bartlett (1932) discovered that memory distortion can occur in such a way that contextual information currently in mind (i.e., information being recalled) can act as a backdrop for the addition of new information.

The integration of new information into an already constructed memory has been shown to occur in both humans and animals. Mnemonic processes are reconstructive in nature, as the act of recalling a memory renders it labile and highly susceptible to modifications (Nader et al., 2000; Debiec et al., 2002; Tse et al., 2007). Memory’s imperfections are not limited to pathological cases, as they are also present in healthy humans, in whom distortions and illusions of memories occur frequently. Such modifications can occur through the incorporation of misinformation into memory from external sources, such as leading questions, deception, and other causes—a phenomenon termed *suggestibility.* They can also occur through the phenomenon of *misattribution*, when retrieved information is assigned to the wrong source. Striking examples abound demonstrating the dramatic instances in which suggestibility and misattribution errors distort memories of crime scenes, childhood events, and traumatic experiences, which were often recalled under interrogation in the court of law or during psychotherapy sessions (Loftus et al., 1978; Schacter and Loftus, 2013).

Interestingly, the activity of the anterior MTL in general and the hippocampus in particular have been positively correlated with the strength of both veridical and false memory recall (Cabeza et al., 2001), thus making the hippocampus an ideal candidate region for interrogating the neuronal conditions that support false memory formation. Amnesic patients with MTL atrophy, likewise, are sometimes less susceptible to false recognition than normal controls (Schacter et al., 1996). Taken together, these results suggest that MTL networks participating in generating episodic imagery are also involved in misremembering imagined events as previously experienced episodes.

Human studies utilizing behavioral and fMRI techniques, however, have not been able to delineate which hippocampal subregions are sufficient for false memory formation, thus necessitating animal models for a more spatially and temporally precise analysis of these neural circuits. While cognitive psychology has greatly enhanced our understanding of false memories through functional neuroimaging and behavioral studies, the underlying neuronal and circuit level processes that enable these cognitive quirks remain vastly unexplored. Few studies to date have utilized rodent models specifically to study the neural substrates underlying false memories. Two studies (McTighe et al., 2010; Romberg et al., 2012) investigated object recognition memory in rats with surgical or pathological perirhinal cortex lesions and found that experimental rats tended to treat novel experiences as familiar, thus leading to the false recognition of objects. Three caveats abound, however: such lesions are difficult to restrict spatially and to reproduce reliably across subjects in terms of volume of brain damaged; these lesions are temporally imprecise; and finally, they do not directly target the cells that participate in forming the engram under investigation.

## Optogenetic inception of a false memory

Building on our previous finding that DG hippocampal cells recruited during learning define an active neural population that is sufficient for memory recall upon subsequent activation (Liu et al., 2012), we asked the following question: can an artificially activated contextual memory engram serve as a conditioned stimulus (CS) and become associated with an unconditioned stimulus (US) to form an artificial CS-US association?

**Figure 2:**
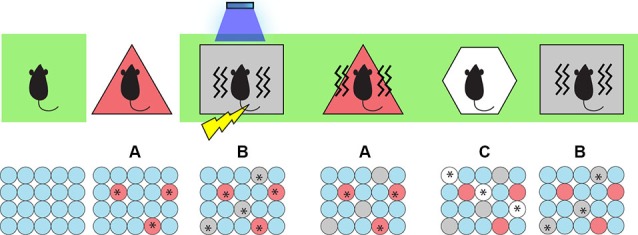
**Inception of a false fear memory.** *Top*: The behavior paradigm for the experimental animals. Animals were kept on Dox post-surgery (green background), then taken off Dox and allowed to explore context A to label active cells with ChR2. Then they were put back on Dox and fear conditioned (lightening symbol) in context B while receiving light stimulation (blue shower symbol) to activate cells representing context A. When they were put back to context A, they showed a false fear memory for A (freezing indicated by wavy lines) where they were never actually shocked. They showed no fear memory for a control context C and a genuine fear memory for context B where they were shocked. *Bottom*: Cellular activity. Red, gray, and white circles indicate neurons representing contexts A, B, and C respectively. Asterisks indicate neurons activated either naturally by contextual exposure or artificially by light stimulation.

To test this, we began by taking the animals off Dox and labeling cells active during the exploration of a neutral context (context A) with ChR2-mCherry. We then put the animals back on Dox and fear conditioned them in a different context (context B) while optically activating the labeled cells involved in encoding the context A. We hypothesized that light-activated context A cells could produce an artificial CS while the mice were simultaneously administered an US to form an artificial associative fear memory. Indeed, when placed back in context A, the experimental group of animals displayed increased freezing levels and hence were freezing to a context in which they were never actually shocked before (Ramirez et al., 2013)—a result that perhaps parallels some types of false recognition memories in humans. Importantly, when placed in a novel context (context C), the animals showed low freezing levels, which indicated that the freezing is context-specific and is not simply a result of generalization (Figure 2).

It is possible that the light-induced activity from context A cells interfered with natural fear memory acquisition of context B. To test this possibility, we re-exposed the trained animals to context B and measured freezing levels across groups. Three findings emerged: (1) the experimental group of animals showed decreased freezing compared to control groups of animals trained in context B alone, suggesting that the activity of context A cells interfered with the animal’s ability to form a representation of context B normally; (2) when given light-on epochs, the experimental group displayed an increase in freezing, which indicated that a natural fear memory (for context B) and an artificially-inducted false fear memory (for context A) can have an additive effect; and (3) in a group of animals that had context A cells labeled with ChR2-mCherry but in which light was omitted during fear conditioning in context B (so that context A cells still represent a neutral context), light-on epochs decreased the freezing responses while re-exposed to context B, suggesting that the activity of cells representing a neutral context A may have a competitive effect on natural fear memory recall for context B.

We could now also probe the behavioral relevance of the DG cells that were artificially associated with an aversive event of high valence (e.g., US). If an artificial CS-US association was generated using our experimental parameters, then activation of these labeled cells should now be sufficient to elicit the associated behavioral output (i.e., freezing). Indeed, when placed in a novel context D and stimulated with light, the experimental group displayed light-induced freezing, suggesting that these activated DG cells have become part of a fear engram as a result of being associated with an US. In a sense, the false memory became a real memory.

To map the downstream brain areas involved in light-induced false memory formation, we measured *c-fos* expression after three different treatments: the false fear memory recall in context A, the natural fear memory recall in context B, and the neutral memory recall in context C. We measured the number of *c-fos* positive cells in the amygdala, which is an essential site for forming fear memories (Rogan et al., 1997). Previous electrophysiological and immunohistochemical studies have shown positive correlations between amygdala IEG activity and freezing levels (Holahan and White, 2004; Knapska and Maren, 2009). Accordingly, we observed that natural fear memory recall in context B and false memory recall in context A elicited similarly robust levels of *c-fos* expression in the basolateral amygdala (BLA) as well as in the central amygdala (CeA), whereas animals exploring context C showed low basal *c-fos* levels. Quantifications revealed that the recall of a natural and false memory both activated 25% of BLA and CeA (Ramirez et al., 2013). These results show that natural fear memory recall and false fear memory recall activate similar proportions of amygdala cells, arguing that both memories recruit similar circuits involved in producing fear memories.

Interestingly, a previous study attempted a similar experimental schedule to generate an artificial memory using pharmacosynthetic methods, and failed to see increased freezing upon re-exposure to either context A or context B alone. Instead, a synthetic memory was observed, which can be recalled only with the presence of both context A (artificially activated by a pharmacosynthetic ligand) and context B (naturally occurring during re-exposure; Garner et al., 2012). Compared to our viral-based optogenetic manipulations, a key difference in their system is that all *c-fos*-expressing cells in the forebrain and midbrain were labeled and activated over the span of minutes to hours with a synthetic ligand. The differences observed here have two implications: the region-specific optogenetic manipulations with millisecond precision, when compared to forebrain-wide pharmacogenetic perturbations that last several minutes, perhaps more reliably recapitulates the endogenous neural activity required for behavioral expression; and, perhaps not all *c-fos*-expressing brain regions are sufficient to elicit the recall of a CS. To further explore this conjecture, we performed the same experiments described above but targeted CA1 instead. We found that 40–70% of CA1 neurons are labeled with ChR2-mCherry in response to context exposure, and we hypothesized that optical activation of such a large population of CA1 neurons, which are also known to utilize highly precise temporal codes to encode information, may not activate a context-specific representation. Indeed, optical stimulation of CA1 engram-bearing cells failed to act as a discrete CS during the presentation of an US to construct an association between the two, as was observed in the DG (Ramirez et al., 2013).

Together, these experiments showed that optical activation of a hippocampal contextual memory engram could act as an artificial CS during fear conditioning to form an artificial CS-US association, or a putative false memory, because the artificial memory never had its contiguous experiences naturally linked. Along similar lines, a recent optogenetic study elegantly showed that pairing lateral amygdala (LA) stimulation—such that activated LA cells substituted as an US—with an auditory CS was sufficient to induce freezing responses later when the CS was presented alone (Johansen et al., 2010), while a second study demonstrated that activating a random population of piriform cortex neurons paired with rewards or shocks could elicit the associated appetitive or aversive behavioral output upon stimulation of the same neurons (Choi et al., 2011). These two studies provide strong evidence that CS and US information can be artificially driven in subpopulations of neurons, and our data expand these findings by providing activity-dependent and context-specific leverage over a defined memory.

While the relationship between our animal model and human false memories remains unclear presently, it does enable future study of memory-updating processes at the level of discrete engrams. Notably, the formation of false memories in humans often occurs as a result of recombining mnemonic elements of discrete experiences into a new, reconstructed memory that is not a veridical representation of the past. These memories are often not *de novo* and require pre-existing memories as a scaffold onto which distinct experiences can be incorporated to update the memory itself (Gershman et al., 2013). Similarly, in our mouse model, our artificial memory is not a *de novo* construction; rather, it is a result of artificially linking a pre-existing memory and an event of high valence. Whether or not the reactivated memory is purely Pavlovian in nature or contains episodic components is a topic currently under investigation.

## Conclusions

In summary, we have shown that hippocampal engram-bearing cell populations underlying previously acquired memories can activate the recall of the associated memories upon subsequent stimulation, and that this activity can form a functional CS, which in turn can be integrated into the simultaneous formation of a discrete fear memory. Our work also suggests that IEG-expressing neurons can form a cellular basis for memory engrams and that these cells have direct, causal relevance in producing memory recall. Identifying the neural underpinnings behind engram formation may yield important clues into the treatment of patients with pathological MTL atrophy, in whom episodic memory is profoundly impaired (Carlesimo and Oscar-Berman, 1992; Fleischman and Gabrieli, 1999).

Since episodic memory is essential to a normal life, an understanding of the normal circuitry underlying hippocampal function is crucial if we are to understand the diseased state in disorders that give rise to episodic memory impairments (Hodges et al., 1990; Storandt et al., 2002; Tamminga, 2013). The findings presented here enable the cellular and functional mapping of memories in different brain regions and the causal dissection of their role in producing associated behaviors.

Indeed, the DG cells we activated to produce recall could also serve as both a technical and conceptual gateway to access memory engrams distributed throughout the brain to produce a variety of behaviors. Such activity-dependent neuronal ensembles are not limited to the DG of the hippocampus, and their roles are not limited to contextual memory either. For example, a recent study found that activating specific ensemble of neurons in the LA was sufficient to induce the recall of an established fear memory (Kim et al., 2014). Similar neuronal ensembles in areas like the prefrontal cortex (PFC), thalamus, BLA, and nucleus accumbens (NAc) have also been shown to play important roles in conditioned addiction (Cruz et al., 2013).

Looking into the future of memory engram research, many challenging questions still remain to be addressed. For example: what is happening at synapse level in these engram-bearing cells (Takeuchi et al., 2013); what is the minimum cell population required to activate a memory (Deng et al., 2013); what occurs to these cells during memory consolidation (Tayler and Wiltgen, 2013) and extinction (Trouche et al., 2013); whether or not defined engram-bearing cells are necessary for memory recall (Drew et al., 2013); and, whether or not similar principles also apply to appetitive memories. From a broader point of view, it is foreseeable that similar engram technologies can be applied to other neuronal circuits to understand complex processes such as anxiety (Felix-Ortiz et al., 2013), depression (Li et al., 2013), and social interaction (Adolphs, 2010; Stowers et al., 2013). On the technology development front, new tools are concurrently being generated to meet these increasing demands. These tools include innovative molecular biology constructs (Mayford, 2013), engineered artificial promoters (Kawashima et al., 2013), novel transgenic animal models (Guenthner et al., 2013; Huang and Zeng, 2013; Sando III et al., 2013), advanced optics (Packer et al., 2013), and cutting-edge nanotools (Alivisatos et al., 2013). Equipped with these powerful tools, memory engram studies will continue to advance our understanding of the brain by causally probing the neuronal basis of learning and memory.

## Author contributions

Steve Ramirez, Susumu Tonegawa and Xu Liu wrote the paper.

## Conflict of interest statement

The authors declare that the research was conducted in the absence of any commercial or financial relationships that could be construed as a potential conflict of interest.
